# Respiratory and mental health effects of wildfires: an ecological study in Galician municipalities (north-west Spain)

**DOI:** 10.1186/1476-069X-10-48

**Published:** 2011-05-21

**Authors:** Francisco Caamano-Isorna, Adolfo Figueiras, Isabel Sastre, Agustín Montes-Martínez, Margarita Taracido,, María Piñeiro-Lamas

**Affiliations:** 1 Consortium for Biomedical Research in Epidemiology & Public Health (CIBERESP). Department of Preventive Medicine, University of Santiago de Compostela. Spain; 2 Culleredo Primary Care Centre, A Coruna, Galician Health Service (Servizo Galego de Saúde - SERGAS), Spain

## Abstract

**Background:**

During the summer of 2006, a wave of wildfires struck Galicia (north-west Spain), giving rise to a disaster situation in which a great deal of the territory was destroyed. Unlike other occasions, the wildfires in this case also threatened farms, houses and even human lives, with the result that the perception of disaster and helplessness was the most acute experienced in recent years. This study sought to analyse the respiratory and mental health effects of the August-2006 fires, using consumption of anxiolytics-hypnotics and drugs for obstructive airway diseases as indicators.

**Methods:**

We conducted an analytical, ecological geographical- and temporal-cluster study, using municipality-month as the study unit. The independent variable was exposure to wildfires in August 2006, with municipalities thus being classified into the following three categories: no exposure; medium exposure; and high exposure. Dependent variables were: (1) anxiolytics-hypnotics; and (2) drugs for obstructive airway diseases consumption. These variables were calculated for the two 12-month periods before and after August 2006. Additive models for time series were used for statistical analysis purposes.

**Results:**

The results revealed a higher consumption of drugs for obstructive airway diseases among pensioners during the months following the wildfires, in municipalities affected versus those unaffected by fire. In terms of consumption of anxiolytics-hypnotics, the results showed a significant increase among men among men overall -pensioners and non-pensioners- in fire-affected municipalities.

**Conclusions:**

Our study indicates that wildfires have a significant effect on population health. The coherence of these results suggests that drug utilisation research is a useful tool for studying morbidity associated with environmental incidents.

## Background

Traumatic events, such as wildfires, can generate psychiatric pathology among the exposed section of the population, i.e., those who are most vulnerable owing to their specific temperamental and neurobiological characteristics, which would act as vulnerability factors ("vulnerability/stress model"). Often, subjects exposed to a traumatic event develop adaptive reactions, which may turn into an acute stress disorder, a common forerunner to post-traumatic stress disorder [1,2].

Smoke is a complex mixture of carbon dioxide, water vapour, carbon monoxide, particulate matter, hydrocarbons and other organic chemicals, nitrogen oxides and trace minerals. Exposure to particulate matter is the main public health threat from short-term exposure to wildfire smoke. In particular, fine airborne particles (PM_2.5 _or particles having an aerodynamic diameter < 2.5 micrometres) constitute the air pollutant with the greatest increase in concentrations during fire events. PM_2.5 _are able to penetrate deep into the respiratory tract, and may cause a whole range of health problems [3,4]. In regulatory guidelines issued by the World Health Organisation (WHO) [5], all particulate matter of a given size class is assumed to be equally toxic regardless of source (biomass as opposed to fossil fuel combustion) or chemical composition. Yet, a number of authors disagree with this and consider that, in view of the currently available evidence in this field, there is a gap in our knowledge [6,7].

Although the adverse effects of urban fine particulate air pollution (PM_2.5_) on respiratory health are well documented, far fewer studies have evaluated the impact of wildfire-generated PM_2.5_, due to the sporadic, unpredictable nature of wildfires and the tendency for air pollution monitors to be situated in predominantly urban areas of highest population concentration [6,8]. Studies that have evaluated the impact of wildfire particulate matter on hospital admissions, emergency department visits or outpatient visits have reported associations with respiratory outcomes (asthma in particular, but also acute bronchitis and chronic obstructive pulmonary disease).

Drug utilisation research was defined by the World Health Organisation in 1977 as, «the marketing, distribution, prescription, and use of drugs in a society, with special emphasis on the resulting medical, social and economic consequences» [9]. Drug utilisation research can be an excellent tool for measuring the morbidity of a population, e.g., by estimating the prevalence of diabetes through the consumption of insulin, oral antidiabetics and other subcutaneously administered antidiabetics [10-12].

Epidemiological assessment of the impact had on disease by natural disasters requires data from the periods before and after the event. As developed countries generally have enough resources for disease surveillance, pre-disaster information is usually obtainable. Post-disaster information may be limited, however, due to severe challenges to the health system posed by the disaster, such as mass migration, damaged infrastructure or saturation of health facilities [13].

Drug utilisation data in Spain are recorded in the billing database managed by the Pharmaceutical Board on the basis of all official prescriptions dispensed. The Spanish pharmaceutical network is made up of many, small, widely distributed pharmacies (each attending to approximately 1,500 inhabitants). By law, pharmacists may only dispense prescription drugs (Rx-only) on production of an official prescription signed by a physician. Where such prescriptions are signed by a National Health Service physician, the customer pays 40% of the retail price of the medication, except in the case of pensioners who are entitled to receive medication free of charge. Once such consumption data have been entered into the country's computerised information systems, they may be accessed and used by health services at any time (pre- and post-disaster).

Little pharmacoepidemiological research has addressed changes in prescription profiles in the wake of a disaster. Some studies have been published on increased prescription rates following an earthquake or terrorist attack [14-16]. There is less information when it comes to seeking a link between fires and drug utilisation [17,18] and almost nothing, if one confines oneself exclusively to forest fires [19].

The main aim of this study was to analyse the respiratory and mental health effects of the August-2006 Galician wildfires, using consumption of anxiolytics-hypnotics and drugs for obstructive airway diseases (DOADs) as indicators.

## Methods

We conducted an analytical, ecological, geographical- and temporal-cluster study. The study unit used was municipality-month (156 municipalities * 27 months, n = 4212) in the Galician provinces of Corunna (*A Coruña*) and Pontevedra.

### Settings

**Figure 1:**
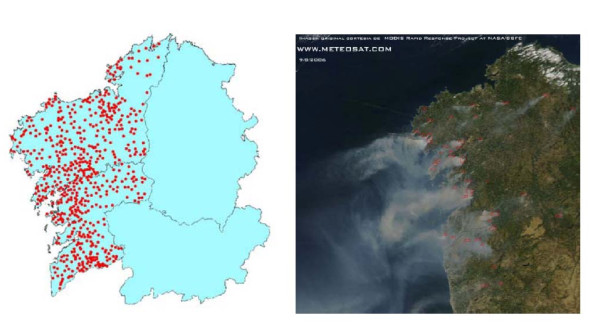
**Wildfires in north-west Spain in August 2006, and satellite photograph**.

During the summer of 2006, a wave of wildfires struck Galicia (north-west Spain), giving rise to a disaster situation in which a great deal of the territory was destroyed [20,21] (Figure 1). Unlike other occasions, the wildfires in this case also threatened farms, houses and even human lives, with the result that the perception of disaster and helplessness was probably the greatest ever experienced in recent years. By the end of that August, a total of 83,000 hectares (7.5% of the territory or 11% of forest surface) in the provinces of Corunna and Pontevedra had been affected by wildfires [22].

In 2006, consumption of benzodiazepine anxiolytics in Spain stood at 46.51 defined daily doses per 1000 inhabitants per day (DDDs), whereas consumption of hypnotics was 22.19 DDDs [23]. Consumption of DOADs is approximately 45 DDDs [24]. Studies show an increase in consumption of around 63% from 1992 to 2006 [24].

### Data-collection

The number of wildfires which had occurred in the respective municipalities in August 2006 was obtained from the Ministry of the Environment [22].

To calculate the consumption, in DDDs, of anxiolytics-hypnotics (N05C and N05B) and DOADs (R03) for each municipality-month, the following three databases were used: 1) the primary health care pharmaceutical billing database managed by the Pharmaceutical Board. This database registers all prescriptions issued by primary care physicians at health facilities managed by the Galician Health Service, which covers approximately 90% of the population. This study was conceived and designed on the assumption that medicines prescribed in any given municipality would be consumed in that same municipality; 2) the Individual Health Card database, which shows the number of inhabitants (population) eligible for Galician Health Service pharmaceutical care, and was used to calculate the indicators; and 3) the Health Ministry's *Nomenclátor Digitalis*, a database that lists all publicly funded medication and contains the fields required to calculate the number of defined daily doses of each drug consumed.

Based on the above information, the wildfires that occurred in August 2006 were allocated to the pertinent municipalities, and the respective DDDs of anxiolytics-hypnotics (N05C and N05B) and DOADs (R03) were allocated to each municipality-month.

Insofar as anxiolytics and hypnotics are concerned, other than their pharmacokinetic differences and strength, it is difficult to find a pharmacodynamic justification for this division of benzodiazepines and equivalents into two therapeutic subgroups. To better understand the higher consumption of benzodiazepines classified as anxiolytics in our health setting, this should be explained along with their conditions of use, characterised in most cases by a low or single nightly dose and short/medium plasma half-life, which is rather similar to treatment with hypnotics. In view of this, we felt it would be very difficult to justify a strict classification of these molecules into the above two groups, and so decided to carry out a joint analysis for both subgroups [23].

### Variables

The independent variable was number of wildfires which had occurred in the municipality in August 2006. Municipalities were thus classified into the following three categories: no exposure (0 to 3 wildfires); medium exposure (from 4 to 10 wildfires); and high exposure (more than 10 wildfires).

Two dependent variables were defined, namely: (1) DDDs of anxiolytics-hypnotics; and (2) DDDs of drugs for obstructive airway diseases. All variables were calculated independently for male non-pensioners, female non-pensioners, male pensioners and female pensioners. Since measuring the 12 months prior to and after social perception of a catastrophe enables the results to be standardised, we calculated these variables for the two 12-month periods pre- and post-August 2006.

### Statistical analysis

We first performed an exploratory analysis of the data, describing the independent (Table 1) and dependent variables (Table 2) and calculating the averages for the pre- and post-fire periods (Table 3). To avoid seasonality, this last calculation was made solely for the months of August and September.

**Table 1:** Distribution of municipalities by exposure: number of municipalities, number of wildfires, mean, and inhabitants covered

	Number of municipalities	Number of wildfires	Mean (95% CI)	Inhabitants
No exposure (0 to 3 wildfires)	47	52	1.11 (0.78 - 1.44)	272,549

Medium exposure (4 to 10 wildfires)	54	362	6.70 (6.12 - 7.29)	964,429

High exposure (11 to 58 wildfires)	55	1076	19.56 (17.09 - 22.04)	799,744

**Table 2:** Consumption of anxiolytics-hypnotics and drugs for obstructive airway diseases

	Anxiolytics - Hypnotics (N05B & N05C) Mean (95% CI)*	Drugs for obstructive airway diseases (R03) Mean (95% CI)*
Total population	100.55 (99.27 - 101.84)	77.27 (76.21 - 78.32)

Men	83.78 (74.09 - 93.48)	92.76 (81.51 - 104.01)

Women	128.77 (115.90 - 141.64)	56.25 (50.60 - 61.89)

Non-pensioners	35.95 (33.87 - 38.04)	21.90 (21.38 - 22.42)

Pensioners	176.60 (170.95 - 182.25)	127.11 (121.14 - 133.08)

Male non-pensioners	23.21 (22.69 - 23.74)	22.46 (21.74 -23.17)

Female non-pensioners	48.70 (47.84 - 49.55)	21.34 (20.58 - 22.11)

Male pensioners	144.36 (141.80 - 146.91)	163.07 (160.20 - 165.93)

Female pensioners	208.84 (204.73 - 212.96)	91.15 (89.03 - 93.27)

* Defined daily doses per 1000 inhabitants/day

**Table 3:** Consumption of anxiolytics-hypnotics and drugs for obstructive airway diseases: consumption pre-wildfire (Aug-Sep 2005) and consumption post-wildfire (Aug-Sep 2006)

	Anxiolytics - Hypnotics (N05B & N05C)		Drugs for obstructive airway diseases (R03)	
	DDDs* mean and (standard deviation)		DDDs* mean and (standard deviation)	

	**Pre-wildfire** **period 2005** **(Aug - Sep)**	**Post-wildfire** **period 2006** **(Aug - Sep)**	**Pre-wildfire** **period 2005** **(Aug - Sep)**	**Post-wildfire** **period 2006** **(Aug - Sep)**

Total population				
Municipalities: no exposure	104.26 (89.10)	107.78 (92.40)	75.93 (71.18)	75.62 (71.55)
Municipalities: med. exposure	90.16 (78.14)	100.71 (81.81)	69.51 (67.87)	73.62 (67.21)
Municipalities: high exposure	89.81 (75.82)	99.37 (79.93)	73.26 (69.12)	73.91 (67.46)

Men				
Municipalities: no exposure	77.19 (67.84)	79.62 (70.87)	95.46 (86.73)	94.38 (87.09)
Municipalities: med. exposure	68.52 (58.71)	76.86 (62.38)	89.93 (82.84)	92.68 (80.58)
Municipalities: high exposure	67.74 (57.14)	74.81 (61.16)	94.80 (84.15)	94.24 (81.38)

Women				
Municipalities: no exposure	131.32 (99.22)	135.95 (102.48)	56.28 (43.02)	56.76 (44.22)
Municipalities: med. exposure	111.70 (88.56)	124.55 (91.57)	49.18 (39.35)	54.48 (42.65)
Municipalities: high exposure	111.89 (85.32)	123.93 (88.62)	51.82 (39.75)	53.58 (40.86)

Non-pensioners				
Municipalities: no exposure	35.34 (25.26)	35.37 (23.29)	19.16 (10.01)	18.61 (9.84)
Municipalities: med. exposure	29.17 (17.23)	33.51 (18.39)	16.21 (7.92)	17.01 (7.47)
Municipalities: high exposure	25.95 (17.19)	33.07 (17.56)	17.86 (8.06)	17.18 (6.21)

Pensioners				
Municipalities: no exposure	173.17 (75.61)	180.19 (77.69)	131.78 (60.47)	132.33 (60.49)
Municipalities: med. exposure	150.86 (67.03)	167.90 (63.30)	122.56 (58.99)	129.98 (50.84)
Municipalities: high exposure	149.68 (63.47)	165.67 (60.55)	128.41 (58.00)	130.64 (51.16)

Male non-pensioners				
Municipalities: no exposure	20.17 (9.75)	19.95 (9.13)	18.81 (9.18)	18.31 (7.48)
Municipalities: med. exposure	18.22 (8.39)	21.17 (8.37)	17.29 (8.08)	17.97 (7.59)
Municipalities: high exposure	18.16 (6.86)	19.93 (7.12)	18.63 (8.17)	17.93 (6.38)

Female non-pensioners				
Municipalities: no exposure	50.52 (27.45)	50.81 (22.93)	19.52 (10.82)	18.91 (11.79)
Municipalities: med. exposure	40.02 (16.88)	45.85 (17.37)	15.13 (7.68)	16.04 (7.24)
Municipalities: high exposure	41.74 (16.31)	46.21 (14.85)	17.10 (7.91)	16.42 (5.97)

Male pensioners				
Municipalities: no exposure	134.21 (50.85)	139.29 (53.08)	171.30 (57.21)	170.45 (59.13)
Municipalities: med. exposure	118.35 (42.27)	132.55 (38.55)	161.90 (56.20)	167.38 (41.52)
Municipalities: high exposure	117.31 (39.40)	129.69 (37.23)	170.28 (50.44)	170.55 (38.90)

Female pensioners				
Municipalities: no exposure	212.12 (76.43)	221.08 (77.07)	92.26 (30.37)	94.21 (30.40)
Municipalities: med. exposure	183.38 (71.47)	203.26 (63.58)	83.23 (26.68)	92.57 (25.39)
Municipalities: high exposure	182.04 (66.53)	201.65 (58.03)	86.55 (26.03)	90.74 (23.06)

* Defined daily doses per 1000 inhabitants/day

We then examined the possible effect of forest fires on the use of anxiolytics-hypnotics and DOADs by means of additive models for time series [25]. The response variables were defined as consumption, in DDDs, of anxiolytics-hypnotics and DOADs, and the explanatory variables considered were municipal level of exposure, and the period, prior and subsequent to the fires, as well as the interaction between the two. To interpret this interaction more intuitively, i.e., of both the estimated coefficients and the confidence intervals, a new variable was created that incorporated both crossings [26]. Each category of this new variable indicated the trend in drug consumption by exposure level before and after the fire (Table 4). Moreover, the time trend in the use of these drugs was fitted using a smooth function, to enable residual autocorrelations to be avoided and the events deemed independent. Thin-plate regression splines were used as trend smoothers, as proposed by Wood [27], and the optimal degrees of freedom were automatically selected using a generalised cross-validation criterion [28]. The analysis was performed for four population groups, due to the fact that consumption, of both anxiolytics-hypnotics and DOADs, is very different for each. The distribution of the consumption of the total population was multimodal and asymmetric. While aggregating data by gender yields a bimodal distribution (two populations), grouping data by occupation yields an asymmetric distribution. When considering four population groups, however, distribution of consumption is Gaussian for each.

**Table 4:** Association between wildfires and consumption of anxiolytics- hypnotics and drugs for obstructive airway diseases: coefficients and *p*-values of additive models for time series

	Anxiolytics - Hypnotics (N05B & N05C)				Drugs for obstructive airway diseases (R03)			
	**Male non-pensioners**	**Female non- pensioners**	**Male pensioners**	**Female pensioners**	**Male non-pensioners**	**Female non-pensioners**	**Male pensioners**	**Female pensioners**

No exposure pre-wildfires (Reference category)	0	0	0	0	0	0	0	0

Medium exposure pre-wildfires	3.15 (<0.01)	-0.43 (0.76)	12.22 (<0.01)	-25.70 (<0.01)	-0.11 (0.88)	-1.14 (0.18)	-12.63 (<0.01)	-8.88 (<0.01)

High exposure pre-wildfires	1.39 (0.05)	0.58 (0.68)	0.82 (0.82)	0.45 (0.93)	-0.34 (0.66)	-1.23 (0.15)	3.76 (0.34)	2.34 (0.36)

No exposure post-wildfires	-1.08 (0.26)	-0.30 (0.87)	3.96 (0.51)	1.42 (0.87)	0.42 (0.83)	-0.29 (0.89)	12.55 (0.15)	9.44 (0.12)

Medium exposure post-wildfires	**2.54** **(0.01)**	-0.36 (0.85)	**21.47** **(<0.01)**	-17.20 (0.05)	1.13 (0.56)	-0.57 (0.79)	-0.56 (0.95)	0.91 (0.88)

High exposure post-wildfires	0.81 (0.40)	1.77 (0.35)	10.42 (0.09)	9.13 (0.29)	-0.25 (0.90)	-1.44 (0.49)	**17.69** **(0.04)**	**11.84** **(0.04)**

For estimating the models, we used the gam function, implemented in the context of the mgcv R package (version 2.10.1) [29].

## Results

Figure 1 depicts wildfire distribution in north-west Spain in August 2006. The main characteristics of the municipalities are broken down by level of exposure in Table 1, and consumption of anxiolytics-hypnotics and DOADs is listed in Table 2. Table 3 shows the post-fire increase in consumption of each group of medicines, with the respective percentage increases according to subject aggregation and municipal exposure level.

The results revealed a higher consumption of DOADs among pensioners during the months after the wildfires, in municipalities affected versus those unaffected by fire (Table 4). The regression coefficients showed that, in municipalities with high exposure after fires, male pensioners' consumption increased by 17.69 DDDs (95% CI: 0.86-34.51) (Table 4). Drawing on the average consumption of the control group for these subjects, 171.89 DDDs, this increase represented a relative increase in consumption of 10.29% (p <0.05). Finally, among female pensioners, the relative increase was 12.09% (p <0.05) (average increase of 11.84 DDDs (95% CI: 6.19-23.49) (Table 4); baseline control group, 97.90 DDDs). There were no significant changes with respect to non-pensioners (Table 4).

Insofar as consumption of anxiolytics-hypnotics was concerned, there was a significant increase among men overall -pensioners and non-pensioners- in fire-affected municipalities (Table 4). Thus, while male non-pensioners registered a relative increase of 12.2% (p <0.05) (average increase of 2.54 DDDs (95% CI: 0.68-4.41) (Table 4); baseline control group, 20.87 DDDs), male pensioners registered a relative increase of 15.88% (p <0.05) (average increase of 21.47 DDDs (95% CI: 9.74-33.21) (Table 4); baseline control group 135.19 DDDs). No effect was observed for women, however.

**Figure 2:**
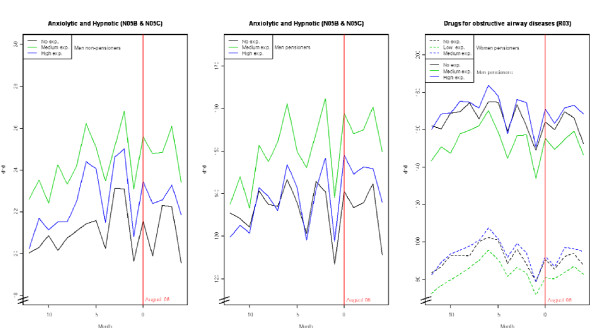
**Consumption of anxiolytics-hypnotics (N05B & NO5C) and drugs for obstructive airway diseases (R03): trends pre- and post-wildfires**.

Figure 2 depicts the monthly trend in consumption for aggregations that displayed significant changes after the wildfires. There is a slight increasing trend especially in the consumption of DOADs among pensioners during the months after wildfires.

## Discussion

Our study indicates that wildfires have a significant effect on population health, in that they are associated with a greater consumption of anxiolytics and hypnotics among men overall, regardless of pensioner status, and with a greater consumption of DOADs among all pensioners, both male and female.

Current evidence strongly suggests that the victims of fire disasters are at increased risk of adverse psychological effects, including post-traumatic stress disorder and depression [30]. Some researchers have indicated that 24% of fire victims display symptoms that would lead to a diagnosis of post-traumatic stress disorder, and 33% show evidence of probable major depression three months after the traumatic event [1]. The source of data affects both the quality and content of the ensuing information, e.g., in one study that investigated the relationship between exposure to a forest fire and mental illness, the authors failed to find an association: this result, which is in contradiction with the literature [31], may be due to the fact that the effect was estimated using physician-visit billing data, which may give rise to possible underestimation due to disease coding errors and other limitations of this variable.

At the outset, neither acute stress disorder nor post-traumatic stress disorder should be treated with medication on a routine basis, without previous evaluation of the subject, and specifically of his/her clinical severity index and the psychological coping strategies involved. Should it be necessary to begin pharmacological treatment for acute stress disorder, the medicines of choice are serotonergic antidepressants. In the short term, however, benzodiazepines may be prescribed to treat anxiety and insomnia, not only in post-traumatic but also in acute stress disorder [32,33].

Accordingly, there is a strong likelihood that many of the people exposed to the Galician forest fires developed acute or post-traumatic stress disorder and that they were initially treated with benzodiazepines. The greater baseline consumption in women might account for the fact that no increase was found in municipalities affected by forest fires (Table 4), given that it is more difficult to find significant increases in cases where baseline consumption is high (Table 2). In men, on the other hand, among non-pensioners and pensioners alike (Table 4), the increase in hypnotics and anxiolytics proved to be significant, and this seems to be coherent with the fact that their baseline consumption was much lower than that of women (Table 2). Our finding of higher baseline consumption of anxiolytics and hypnotics among women versus men (Table 2) coincides with data reported by other researchers [34]. The 2006 National Health Survey (*Encuesta Nacional de Salud 2006*) [35] reveals that the percentage of persons that had consumed, "tranquilisers, relaxants and sleeping pills", i.e., benzodiazepines, within the preceding two weeks was considerable, and higher in women than in men (17.5% versus 10.18% respectively). This higher prevalence of consumption among women may be due to a higher degree of psychiatric morbidity [33-37], a lower tolerance to stress [38], a tendency to seek help for psychiatric problems more often [36], a higher degree of acceptance of treatment [37] and a higher probability of being frequent attenders in general practice [35,39,40] than their male counterparts, all of which is linked to greater consumption of psychopharmaceutical drugs [33].

In our study, DOAD consumption increased significantly among pensioners (men and women) (Table 4). This result is coherent with the fact that a recent stressful event, and emotional stress in general, constitutes a risk factor for poor asthma control [41]. It is also line with the conclusions reached in the most significant study published to date regarding the impact of a large-scale forest fire on cardio-respiratory morbidity, namely, that the strongest association between exposure to particles smaller than 2.5 micra and hospital admissions due to asthma is to be found in the population aged 65-99 years (an increase of 10.1%) [8]. In this respect, it should be noted that 77% of all pensioners in Spain are over the age of 65 years [42,43].

Our study has the following three main limitations: (1) it has been argued that the validity of these types of studies can be questioned [10,44], due to underdiagnosis [10], drug non-specificity for a single pathology [9,10,44], existence of patients diagnosed for non-pharmacological treatments [10], physician-prescribed medication that is not dispensed to patients in pharmacies (non-fulfilment) [45,46] and use of prescribed medication under conditions other than those established in the drug prospectus inserted in the package. Nevertheless, these limitations vary according to the source of the data and may not be significant when the trend in consumption is analysed and a control group is available; (2) measurement of the dependent variable assumes that all medication sold in pharmacies within a given ecological unit are consumed by the population from that same unit, an assumption that may not necessarily be correct. In contrast, a cohort study would enable a specific consumption of medicines to be allocated to each subject (optimum design). Even so, we consider that our assumption may prove to be a good approximation, bearing in mind that there is a very uniform, widespread network of pharmacies in Galicia. In this particular case, therefore, the validity of a cohort study would be similar to that of an ecological study; and, (3) the primary healthcare database does not include prescriptions issued by hospitals, civil servant mutual insurance companies and private doctors. However, this limitation would mainly affect the study's descriptive results: its analytical results would be affected to a lesser extent, and primary healthcare prescriptions for hypnotics and anxiolytics have been estimated to represent around 87% of the total [47], most of which is dispensed by medical prescription [48].

## Conclusions

Our study indicates that wildfires have a significant effect on population health. The coherence of these results suggests that drug utilisation research is a useful tool for studying morbidity associated with environmental incidents.

## List of Abbreviations

DDDs: defined daily doses per 1000 inhabitants/day; DOADs: drugs for obstructive airway diseases

## Competing interests

The authors declare that they have no competing interests.

## Authors' contributions

FC and AF conceived the study and drafted the manuscript. AM and MT participated in its design and co-ordination, and helped draft the manuscript. MP performed the statistical analyses. IS wrote the final manuscript. All authors read and approved the final manuscript.
